# Cutaneous Cryptococcoma in a Patient on TNF-α Inhibition

**DOI:** 10.3390/jcm2040260

**Published:** 2013-11-22

**Authors:** Rui M. Gomes, Dean R. Cerio, Cyrus Loghmanee, Justin McKinney, Mili Patel, Janeen Miraglia, Manal Yousef-Bessler, Jonathan H. Zippin, Audrey N. Schuetz, Paulo Bandeira Pinho

**Affiliations:** 1 PASE Healthcare, PC, 225 Millburn Avenue, Suite 303, The Common, Millburn, NJ 07041, USA; E-Mails: ruigomesmd@gmail.com (R.M.G.); mpatel@pasehealthcare.com (M.P.); jmiraglia@pasehealthcare.com (J.M.); pinhomd@pasehealthcare.com (P.B.P.); 2 East Coast Advanced Plastic Surgery, 79 Hudson Street, Suite 700, Hoboken, NJ 07030, USA; E-Mails: dean@ecaplasticsurgery.com (D.R.C.); cyrus@ecaplasticsurgery.com (C.L.); 3 Saint Joseph’s Regional Medical Center, 703 Main Street, Paterson, NJ 07503, USA; E-Mail: jmckinne@nyit.edu; 4 Infectious Disease Center of New Jersey, 22 Old Short Hills Road, Suite 106, Livingston, NJ 07039, USA; E-Mail: manalmd@aol.com; 5 Weill Cornell Medical Center, New York Presbyterian Hospital, 525 East 68th Street, New York, NY 10021, USA; E-Mails: jhzippin@med.cornell.edu (J.H.Z.); ans9112@med.cornell.edu (A.N.S.)

**Keywords:** panniculitis, *Cryptococcus neoformans*, adalimumab, TNF-α inhibitor, fluconazole

## Abstract

An 87-year old Caucasian male with past medical history of rheumatoid arthritis (RA) and chronic kidney disease presents with left hand erythema, pain, tenderness, induration and edema. Clinically, these hand findings began proximal to the metacarpo-phalangeal joints and extended to the distal wrist. He was noted to have ipsilateral axillary lymph node enlargement but denied any constitutional signs or symptoms. Laboratory markers of inflammation were poor prognostic indicators due to relatively active RA, the use of chronic daily glucocorticoids and weekly adalimumab use. Oral antibiotics were administered with limited success leading to a skin biopsy which reported a hematogenously disseminated fungal panniculitis; cultures grew *Cryptococcus* *neoformans*, however, serum cryptococcal antigen was negative. With initial fluconazole treatment, skin findings and lymphadenopathy improved gradually over the next six months. However, the patient’s improvement stagnated and his condition reverted back to the state of initial presentation.

## 1. Introduction

An 87-year old Caucasian male, resident in the United States for over 50 years, with past medical history of rheumatoid arthritis (RA) and chronic kidney disease presents with left hand erythema, pain, tenderness, induration and edema. Clinically, these hand findings began proximal to the metacarpo-phalangeal joints and extended to the distal wrist. Erythema was 9 cm with central induration and swelling measuring 2 cm. The patient had no limitation of motion due to swelling, pain, neurologic or vascular compromise. He was noted to have ipsilateral axillary lymph node enlargement but denied any constitutional signs or symptoms. Laboratory markers of inflammation were poor prognostic indicators due to relatively active RA, the use of chronic daily glucocorticoids and weekly adalimumab use. The patient had traveled to Portugal two years ago where he had no contact with farm animals. After withholding adalimumab and treating with escalating spectrum of antibiotics over a month with minimal improvement, an MRI was performed showing infiltration of the ulnar aspect subcutaneous fat without evidence of osseous damage. Therefore, oral antibiotics were continued with limited success leading to a skin biopsy which reported a hematogenously disseminated fungal panniculitis. Both the bacterial and the fungal cultures of the skin biopsy grew *Cryptococcus neoformans*. On fungal culture, sparse growth of yeast-like colonies was obtained on week three of culture. The yeast grew on Sabouraud dextrose agar at 30 °C. Round, narrow-budding yeasts were seen on wet preparation of the colonies. Urease was positive, and a caffeic acid disk test was positive. The Vitek2 YST card (bioMérieux, Durham, NC, USA; code number 21343) identified the yeast as *C. neoformans*/*C. gattii*. Differentiation between *C. neoformans* and *C. gattii* was not performed on this isolate, as that practice was only performed for immunocompetent patients with unexplained cryptococcosis. Serum cryptococcal antigen was negative. Antifungal drug susceptibility testing was not requested since it is known that *Cryptococcus neoformans* is almost universally susceptible to fluconazole, especially when the patient has no history of previous fluconazole administration. With initial fluconazole treatment, skin findings and lymphadenopathy improved gradually over the next six months. However, the patient’s improvement stagnated and his condition reverted back to the state of initial presentation. Given this, his hand was explored surgically and a cryptococcoma was excised in its entirety (histopathologically-confirmed diagnosis of *Cryptococcus neoformans* infection). The patient was treated with fluconazole for a total of six months after the surgical exploration and within a few post-operative months was pain and swelling free and enjoyed his pre-infected functional state.

## 2. Results and Discussion

*Cryptococcus* species are encapsulated yeasts that infect humans and were first described in 1894. Infection with cryptococcal species presents across a wide spectrum usually as meningoencephalitis and pneumonitis, with cutaneous infections appearing more uncommonly. Soft tissue cryptococcal infections include cellulitis, necrotizing fasciitis, and cryptococcomas [1]. 

In humans, TNF-α is responsible for macrophage and phagosome activation, it differentiates monocytes into macrophages and leads to macrophage recruitment to sites of infection [2]. Given the fact that cryptococcal species are encapsulated yeasts, eradication occurs primarily through macrophage phagocytosis. In this patient, inhibition of TNF-α with adalimumab led to diminished macrophage function and increased susceptibility to opportunistic infection.

Although no cases of cryptococcosis have been detected in 10,050 treated patients in the United States post-marketing database for adalimumab, other cases of invasive cryptococcosis in a patient receiving adalimumab have been documented and reinforce the relationship between TNF-α antagonists and the emergence of severe opportunistic infections [3].

*Cryptococcus* *gattii* primarily occurs in immunocompetent hosts whereas 90% of *Cryptococcus* *neoformans* infections occur in immunocompromised hosts [4]. Differentiation of *C. neoformans* from *C. gattii* may be performed via either sequencing or through use of canavanine glycine bromothymol blue (CGB) agar. However, at this point in time, it is not routine for laboratories to perform such differentiation. Differentiation can be performed for isolates cultured from either immunocompetent patients or those patients with significant travel history to the Pacific Northwest region of Canada and the United States. Our patient did not have risk factors at the time for *C. gattii* and so was not tested. Although clinical human cases of *C. gattii* are still unusual in areas outside of tropical and subtropical climates as well as the Pacific Northwest, scientists are discovering ecologic niches in temperate climates of Europe and elsewhere which may contribute to transmission of this organism [5]

Treatment of patients with mild to moderate cryptococcal infections consists of fluconazole for 6–12 months as first line therapy. Other acceptable regimens include itraconazole, voriconazole, posaconazole and amphotericin B. Surgery remains a viable option for those patients who cannot tolerate antimicrobial therapy or those that fail to respond, as was the case in this patient [5].

## 3. Conclusions

This case demonstrates the awareness required by physicians to identify uncommon pathogens found in skin infections, especially in those who are immunocompromised [5]. While not widely reported, TNF-α inhibition with adalimumab provides a clinical environment for development of cutaneous cryptococcal disease. A high degree of suspicion for these pathogens is necessary when assessing immunosuppressed patients with skin lesions that fail to improve despite conventional therapy [6].
